# Clinical Characteristics and Management of Snake Bite Injuries in the Jerusalem Area

**DOI:** 10.3390/jcm12124132

**Published:** 2023-06-19

**Authors:** Itai Gross, Aus Maree, David Rekhtman, Waseem Mujahed, Saar Hashavya, Jacob Assaf

**Affiliations:** 1 Department of Pediatric Emergency Medicine, Hadassah Medical Center, Jerusalem 91120, Israel; crazy_jek@yahoo.com (D.R.); saarha@gmail.com (S.H.); 2 Department of Pediatrics, Hadassah Medical Center, Jerusalem 91120, Israel; aus.maree10@gmail.com; 3 Department of Emergency Medicine, Hadassah Medical Center, Jerusalem 91120, Israel; drwaseemmuj@gmail.com (W.M.); jassaf@hadassah.org.il (J.A.)

**Keywords:** snake bite, *Daboia palaestinae*, *Vipera palaestinae*, *Atractaspis engaddensis*, *Echis colorata*, children

## Abstract

Venomous snake bites can constitute medical emergencies, and without immediate care may be life-threatening. This study describes the characteristics and management of patients suffering from snake bite injuries (SNIs) in the Jerusalem area. A retrospective analysis of all patients who were admitted to the Hadassah Medical Center emergency departments (EDs) due to SNIs between 1 January 2004 and 31 March 2018 was conducted. During this period, 104 patients were diagnosed with SNIs, of whom 32 (30.7%) were children. Overall, 74 (71.1%) patients were treated with antivenom, 43 (41.3%) were admitted to intensive care units, and 9 (8.6%) required treatment with vasopressors. No mortality was recorded. On ED admission, none of the adult patients presented with an altered mental state compared to 15.6% of the children (*p* < 0.00001). Cardiovascular symptoms were observed in 18.8% and 5.5% of the children and adults, respectively. Fang marks appeared in all of the children. These findings underscore the severity of SNIs and the differences in clinical presentation between children and adults in the Jerusalem region.

## 1. Introduction

Venomous snake bites can constitute medical emergencies, and without immediate care can be life-threatening. Snake bites are the cause of substantial mortality and morbidity worldwide, especially in rural areas [1,2,3,4,5]. Eight species of venomous snakes are endemic to Israel, three of which are considered dangerous to humans: *Daboia* (*Vipera*) *palaestinae* (previously known as *Vipera palaestinae*), *Echis colorata* and *Atractaspis engaddensis. Daboia palaestinae* is the most common venomous snake in Israel. It is found in the central and northern regions and neighboring countries, and accounts for 100–300 bites annually in Israel. *Echis colorata* is found in all parts of Israel, the Middle East and Egypt. It is usually found in less-populated areas. Its venom causes a severe systemic reaction including afibrinogenemia and coagulopathies. *Atractaspis engaddensis* is considered the rarest of all venomous snakes in Israel, and is found solely in the south. Its venom has strong cardiotoxic activity. There is currently no available antivenom for *Atractaspis engaddensis* [6,7,8].

The severity and effects of a snake bite depend on a number of factors, but first and foremost the type of snake and the amount of venom injected into the victim [2]. Only a few studies on snake bites in children have been published [3,8,9,10,11,12,13] and there are no studies comparing children and adults in Israel. This study thus fills this gap by characterizing the similarities and differences between pediatric and adult snake bites in this region.

## 2. Methods

A retrospective analysis of the data collected on all patients diagnosed with venomous snake bites and admitted to the Hadassah Medical Center in Jerusalem, Israel from 1 January 2004 to 31 July 2018 was conducted. The data obtained from these records consisted of demographics (age and gender), history (a description of the snake bite, location, time of year, description of the snake, pain level, and affected organs), symptoms, physical examination findings, vital signs, laboratory work, and hospitalization data (length, admission in the intensive care unit (ICU), and treatment). To better understand the characteristics of children suffering from snake bites, we compared children under the age of 18 years to adults. 

Antivenom was given according to local protocol to every patient with systemic symptoms or a severe local reaction including progressive swelling/clinical deterioration. A second dose of antivenom was given in cases of systemic deterioration or worsening of the local reaction. The type of antivenom depended on the suspected snake: *Daboia* (*Vipera*) *palaestinae*/*Echis coloratus* antiserum (equine source; Kamada Ltd., Beit Kama, Israel).

This study was approved by the hospital institutional review board and a consent waiver was obtained (approval number 0433-18-HMO). 

### Statistical Analysis

The data are presented as the mean and standard deviation (SD) or the number of patients and percentages. A chi-square test was used to compare proportions and a Student’s *t*-test was used to compare continuous parametric variables. A *p* value of 0.05 or less was considered statistically significant. Statistical analyses were performed using SPSS version 21.0 (Statistical Package for Social Science, Chicago, IL, USA).

## 3. Results

During the study period, 104 patients were diagnosed with snake bite-related injuries. The average age (±SD) at presentation was 28.6 (±18.2) years; 32 (30.7%) of the patients were children (aged < 18 years). In the pediatric group, there were more females than in the adult group (43.8% vs. 20.8%; *p* = 0.01; Table 1). Most snake bites occurred during the summer (47.2%) in adults and in the autumn in the case of children (46.9%). Cardiovascular (including age-adjusted tachycardia, bradycardia and hypotension) symptoms were more common in the pediatric group (18.8% vs. 5.5%; *p* = 0.03) as well as altered mental state, as defined by a Glasgow Coma Scale score of ≤14 (15.6% vs. 0%; *p* < 0.00001; Table 1). 

Length of hospitalization was 3.6 ± 3.4 days; 31.7% of patients were hospitalized in the ICU and 53.8% were treated with antivenom. None of the children had an allergic reaction post-antivenom treatment compared to 21% of the adults (*p* = 0.005). There was a complete recovery after initial treatment in 86.8% of the patients and on discharge in 95.2% of the patients. During the study period, there were no cases of mortality (Table 2). 

Upon physical examination, 100% of the children and 91.7% of the adults had fang marks (*p* = 0.09), and children were more likely to develop local ecchymosis at the site of the wound (21.9% vs. 4.2%; *p* < 0.01). A comparison of laboratory tests showed that there were fewer cases of age-adjusted renal failure in children (3.1% vs. 18.1%; *p* = 0.04). No other clinically important statistically significant differences were found between the two groups (Table 3).

## 4. Discussion

Snake bite-related injuries are a common cause of morbidity and mortality worldwide [1,2,3,4,5]. The current study presented the characteristics of snake bite injuries in Israel. The findings, which are appliable to Israel and the adjoining regions, revealed significant differences in children suffering from snake bites compared to adults; although, they both underwent a similar clinical course with similar mortality rates (none) and morbidity. Although the amount of venom that a snake injects into its prey is not assumed to be different for a child or an adult victim, there were no differences in the course of hospitalization, treatment, or prognosis between the two groups (Table 2). However, 15.6% of children suffered from an altered mental state (three times higher than adults), and more children had an age-adjusted low blood pressure (18.8% vs. 4.2%; *p* = 0.01 in children and adults, respectively). Some of these children had no history of snake bites and the diagnosis was based on the discovery of fang marks upon physical examination. A similar observation was reported in another study on pediatric *Daboia* (*Vipera*) *palaestinae* snake bites where 18% of the children were drowsy upon presentation and 95% had fang marks [8]. In another study, 26% of all pediatric victims had neurological symptoms [10]. In a study comparing envenomed children and adults, 22% of the children suffered from hypotension compared to only 11.4% of the adults (*p* = 0.01). Given all of the above, in addition to the fact that evidence of a snake bite was found in all of the children in this study, in cases of pediatric unexplained altered mental state, the physician should consider the eventuality of a snake bite and perform a thorough physical examination. One possible explanation for the higher percentage of altered mental state in children and their lower blood pressure could be a state of shock caused by the higher amount of venom per Kg in children. In the current study, although the children’s initial presentations were more severe, it did not result in more complicated courses of hospitalization or worse prognoses, which is probably due to the relatively timely treatment provided. Another explanation for the overall similarity in course of hospitalization and prognosis, could be that the toxicity of the common venomous snakes in our region is not as severe as other snake types as shown before [5]. This fact was demonstrated by 72% of the patients having no systemic symptoms at all, and no cases of mortality in our cohort. It is possible that, although the initial presentation was severe, due to the relatively mild toxicity, children were able to overcome the envenomation. To the best of our knowledge, no similar findings have been reported elsewhere.

In the current study, none of the children who were administered antivenom developed a significant allergic reaction, unlike in a much earlier study conducted in Israel by Paret et al. in which 8% of children developed a skin rash; although, no major allergic reactions were noted and no use of adrenaline was reported [8]. Other studies, in other parts of the world where different antivenoms have been used, have indicated allergic reactions in 14% to 45.5% of children [10,13,14].

We found that most snake bites occurred during the summer (47.2%) in adults and in the autumn in the case of children (46.9%). This finding could be explained by the fact that autumn is the holiday season in Israel when most of the schools’ and youth movements’ outdoor activities and field trips take place.

## 5. Limitations

There are several limitations to this study which deserve attention. First, its retrospective nature resulted in incomplete documentation. The type of snake was uncertain in most cases; a fact that makes generalization of the conclusions limited. In addition, this study was only conducted in two centers in Jerusalem, which may have led to referral bias. Although considered the largest medical facilities in our area, we cannot exclude re-admissions of discharged patients to other medical centers. Finally, the data and conclusions only apply to local snake species, thus limiting the generalizability of the findings to countries in the Middle East.

## 6. Conclusions

In Israel, and more generally in the Middle East, children may present with different clinical signs than adults after snake envenomation. Altered mental state is an important marker of envenomation. Given the high incidence of ecchymosis and fang marks, which were found to be more prominent in children, a meticulous, oriented physical examination of all children with altered mental state and low blood pressure is advisable.

## Figures and Tables

**Table 1:** Comparison of demographics, history and symptoms in children (aged < 18) vs. adults treated for a venomous snake bite.

	Total (n = 104)	Children (n = 32)	Adults (n = 72)
Age, mean (SD), years	28.6 (18.2)	10.1 (4.8)	36.8 (15.7)
Gender, number of females, n (%)	29 (27.9%)	14 (43.8%)	15 (20.8%)
Time from bite to arrival at the emergency department, mean (SD), hours	1.46 (1.5)	1.08 (0.9)	1.66 (1.71)
Gastrointestinal symptoms, n (%)	16 (15.4%)	5 (15.6%)	11 (15.3%)
Central nervous system symptoms, n (%)	2 (1.9%)	0 (0%)	2 (2.8%)
Respiratory symptoms, n (%)	7 (6.7%)	1 (3.1%)	6 (8.3%)
Cardiovascular symptoms, n (%)	9 (8.6%)	6 (18.8%)	4 (5.5%)
No systemic symptoms, n (%)	75 (72.1%)	23 (71.9%)	52 (72.2%)
Impaired consciousness, n (%)	5 (4.8%)	5 (15.6%)	0 (0%)

**Table 2:** Comparative hospitalization profile for children (aged < 18) vs. adults treated for venomous a snake bite.

	Total (n = 104)	Children (n = 32)	Adults (n = 72)	*p* Value
Length of hospitalization, mean days (SD)	3.6 (3.4)	3.5 (2.8)	3.6 (3.6)	0.91
ICU hospitalization, n (%)	32 (31.7%)	11 (34.4%)	21 (30.6%)	0.7
Treatment with antivenom, n (%)	56 (53.8%)	18 (56.3%)	38 (52.8%)	0.74
Treatment with antihistamines, n (%)	39 (37.5%)	11 (34.4%)	28 (38.9%)	0.66
Treatment with steroids, n (%)	26 (25%)	5 (15.6%)	21 (29.2%)	0.14
Treatment with antibiotics, n (%)	37 (35.6%)	13 (40.6%)	24 (33.3%)	0.47
Tetanus vaccination, n (%)	20 (19.2%)	5 (15.6%)	15 (20.8%)	0.53
Complete recovery post-initial treatment, n (%)	66/76 (86.8%)	19/21 (90.4%)	47/55 (85.5%)	0.57
Allergic reaction post-antivenom treatment, n (%)	8/56 (14.3%)	0/18 (0%)	8/38 (21%)	NA
Need for vasopressors, n (%)	7 (6.7%)	2 (6.3%)	5 (6.9%)	0.91
Need for blood products, n (%)	3 (2.9%)	2 (6.3%)	1 (1.4%)	0.17
Complete recovery post-hospitalization, n (%)	99 (95.2%)	30 (94%)	69 (95.8%)	0.69

**Table 3:** Comparison of physical examination findings in children (aged < 18) vs. adults treated for a venomous snake bite.

	Total (n = 104)	Children (n = 32)	Adults (n = 72)	*p* Value
Desaturation, n (%)	6 (5.8%)	1 (3.1%)	5 (6.9%)	0.44
Abnormal pulse *, n (%)	14 (13.5%)	7 (21.9%)	7 (9.7%)	0.09
Low blood pressure *, n (%)	9 (8.7%)	6 (18.8%)	3 (4.2%)	0.01
Bite signs, n (%)	98 (94.2%)	32 (100%)	66 (91.7%)	0.09
Swelling, n (%)	94 (90.4%)	30 (93.8%)	64 (88.9%)	0.43
Redness, n (%)	65 (53.8%)	18 (56.3%)	38 (52.8%)	0.74
Ecchymosis, n (%)	10 (9.6%)	7 (21.9%)	3 (4.2%)	<0.01

* In comparison to the age-adjusted reference (Harriet Lane Handbook 21st ed.).

## Data Availability

The data presented in this study are available on request from the corresponding author.

## Abbreviations

ED—emergency department; ICU—intensive care unit; WBC—white blood count; CRP—C-reactive protein; ESR—erythrocyte sedimentation rate.
